# Get insight into the cause of death distribution and epidemiology of penile squamous cell carcinoma: A population‐based study

**DOI:** 10.1002/cam4.4614

**Published:** 2022-03-03

**Authors:** Xiangpeng Zhan, Luyao Chen, Ming Jiang, Bin Fu

**Affiliations:** ^1^ Department of Urology The First Affiliated Hospital of Nanchang University Nanchang China

**Keywords:** cause of death, epidemiology, penile squamous cell carcinoma, race, SEER, trend

## Abstract

**Background:**

Penile squamous cell carcinoma (PSCC) survival had no significant improvement since 1990 in the United States. This study aims to get insight into the changing trend and distribution of death causes of PSCC. The epidemiology of PSCC is also investigated.

**Methods:**

The Surveillance, Epidemiology, and End Results (SEER) (1992–2018) database is utilized to get patients diagnosed with penile squamous cell carcinoma. The trend of incidence, distribution of age, changing trend and distribution of death cause, and survival outcome are analyzed for all PSCC patients and each race.

**Results:**

Three thousand four hundred and twenty‐three male patients with PSCC are enrolled in our study. The age‐adjusted incidence rate of the white has a slight increase (Annual percent change [APC] = 0.647%). American Indian/Alaska Native men have the highest average annual incidence, while Asian /Pacific Islander men have the lowest. PSCC patients aged 70–80 are the most common, and patients over 80 years have the highest 3‐year (50%) and 5‐year (63.93%) mortality rate. Non‐cancer disease, especially circulatory system disease, is the most common cause of death, whereas the proportion of patients who died of PSCC significantly increased from 21.17% (1992–2001) to 41.3% (2012–2017) in PSCC patients (*p* < 0.001). These results have not changed significantly when we only focus on primary PSCC without previous malignant tumors. Hispanics are shown better overall survival than non‐Hispanic White and non‐ Hispanic Black men. (*p* < 0.001) No statistical differences in cancer‐specific survival are observed (*p* = 0.15).

**Conclusion:**

The current study provides essential initial data regarding the presentation and clinical outcomes of PSCC patients. Notably, non‐cancer disease, especially circulatory system disease, is the more common cause of death than PSCC. However, the proportion of patients who died of penile squamous cell carcinoma has a relatively significant increase in recent years. The increasing trends in the advanced stage of PSCC patients might account for this change.

## Graphical abstract

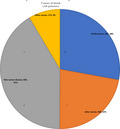

## 1 INTRODUCTION

Penile squamous cell carcinoma (PSCC) is a rare disease, which presents approximately 0.4%–0.6% of all male malignant neoplasms in Europe and America. The proportion is significantly up to 10% in developing countries like Asia, Africa, and South America.^1^, ^2^, ^3^ Meanwhile, it is also an aggressive disease with a relatively high mortality rate. In the United States, about 2320 new PSCC patients are diagnosed, and 380 deaths are owing to it in 2018.^2^ Risk factors confirmed associated with PSCC include human papillomavirus (HPV) infection, smoking, circumcision status, and lower socioeconomic status.^4^, ^5^ Race/ethnicity is gradually recognized as a determinant of penile cancer incidence and mortality.^6^, ^7^ A retrospective study suggests that White Hispanics have higher incidence rates when compared with Alaska Native/American Indians and Black men, even if they are from the same country.^7^ Meanwhile, a study based on the American population indicates that African‐American men have worse overall survival than Caucasian American men.^1^ The etiology for these discrepancies is still not well understood, but some ideas proposed are due to circumcision practices, rates of HPV infection, and economic level of living.^8^, ^9^

The majority of penile cancer is diagnosed as squamous cell carcinomas (SCC).^10^ Over the last decade, the surgery of penile‐preserving has been more common. It is a kind of surgery reported to achieve an excellent balance of cancer control and relatively satisfactory functional outcomes, although these results are limited in some retrospective studies.^11^, ^12^, ^13^, ^14^ In addition, of patients with pelvic lymph node‐positive, more prolonged overall survival was observed in patients undergoing adjuvant chemotherapy.^15^ However, no significant improvement in survival was obtained in the USA since at least 1990 based on the data from the SEER database.^16^ The other co‐morbidities also seem common for PSCC patients considering patients diagnosed with PSCC at an older age––on average at age 60.^17^ In this case, the other cause of death will likely play a more and more important role in death cause of PSCC patients, when compared to cause of PSCC in the coming years.

Up to our knowledge, the cause of death distribution in PSCC patients has not been specifically investigated in the United States. Moreover, changing trends in cause of death distribution have not yet been explored. Due to the lower population incidence, current data on PSCC are still limited to retrospective observational studies. In the present study, we searched the Surveillance, Epidemiology, and End Results (SEER) database to enroll patients diagnosed with PSCC. The primary purpose of our research is to explore the cause of death distribution of PSCC, and changing trends by era are also investigated. Meanwhile, we investigated the trend of age‐adjusted incidence rates, characteristics of age distribution, survival outcomes, and mortality rate stratified by race to get insight into the epidemiology of penile squamous cell carcinoma.

## 2 MATERIALS AND METHODS

### 2.1 Study population and data sources

All patients enrolled in this study are from the Surveillance, Epidemiology, and End Results (SEER) database, which collected data on cancer incidence and mortality from the U.S. population since 1973. The data from SEER are publicly available and de‐identified. Therefore, institutional review board approval is not required. We search the case listing from the dataset of *Incidence ‐ SEER Research Plus Data, 13 Registries, Nov 2020 Sub (1992–2018)—Linked To County Attributes—total U.S*. First, we enroll all patients diagnosed with penile squamous cell carcinoma according to the list of site recode ICD‐O‐3 from 1992 to 2017. In addition, we excluded 27 patients with unknown race and 87 patients with the cause of death not recorded because they failed to match with the purpose of research.

### 2.2 Cause of death data

The underlying cause of death from the death certificate is grouped into a recode in the SEER database. The SEER program used the International Classification of Diseases, Ninth Edition (ICD‐9), to code patients who died from 1979 to 1998, while ICD Tenth Edition (ICD‐10) is applied to code patients who died after 1998.

To facilitate analysis, we divide the cause of death into four primary categories: the death of PSCC, death of other cancers, death of non‐cancer disease, and non‐disease cause (accidents and adverse effects, congenital anomalies, homicide, and legal intervention, other cause of death). In addition, we have subdivided the death of other cancers and the death of non‐cancer disease on the basis of systemic diseases. Death of other cancers mainly included leukemia, digestive system cancer, genitourinary system cancer, respiratory system cancer, and other cancer. Death of non‐cancer disease included nervous system disease, circulatory system disease, digestive system disease, respiratory system disease, diabetes mellitus, genitourinary system, and other causes (Tables 1 and 2).

**TABLE 1:** Population characteristics of persons diagnosed with penile squamous cell carcinoma in the Surveillance, Epidemiology, and End Results Program (SEER) (1992–2018)

Characteristics	Overall US SEER (*n* = 3423)	Non‐hispanics US (*n* = 2735)	Hispanics US (*n* = 688)
Age (year)			
Mean	67.03	68.83	59.58
Median (25th–75th percentile)	67.5 (57.5–77.5)	72 (72–77.5)	62 (47.5–72)
<40	122 (3.5%)	38 (1.4%)	84 (12.2%)
40–50	307 (8.9%)	179 (6.5%)	127 (18.5%)
50–60	563 (16.4%)	436 (15.9%)	126 (18.3%)
60–70	806 (23.4%)	671 (24.5%)	134 (19.5%)
70–80	914 (26.5%)	783 (28.6%)	127 (18.5%)
>80	731 (21.2%)	628 (23.0%)	90 (13.1%)
Year of diagnosis			
1992–1996	518 (15.13%)	435 (15.9%)	83 (12.06%)
1997–2001	565 (16.5%)	471 (17.22%)	94 (13.66%)
2002–2006	593 (17.32%)	473 (17.29%)	120 (17.44%)
2007–2011	761 (22.23%)	601 (21.97%)	160 (23.26%)
2012–2017	986 (31.82%)	755 (27.62%)	231 (33.58%)
Median survival time (month) **(**25th–75th percentile)	44 (14–100)	45 (15–102)	67.07 (12–93.75)
Mean survival time (month)	67.03	68.02	63.12
Median age at death (year)	67.5	72	57.5
3‐year mortality rate	1091 (31.87%)	885 (32.36%)	206 (29.94%)
5‐year mortality rate	1378 (40.26%)	1136 (41.55%)	242 (35.17%)
Died from all causes	2016 (58.9%)	1696 (62.01%)	320 (46.51%)
Died from Penile cancer	562 (16.42%)	429 (15.69%)	133 (19.33%)
Died from other disease	1282 (37.45%)	1113 (40.69%)	169 (24.56%)
Cause of other cancer	448 (13.09%)	389 (14.22%)	59 (8.58%)
Leukemia	36 (1.05%)	35 (1.28%)	1 (0.015%)
Digestive system cancer	72 (2.1%)	66 (2.41%)	6 (0.087%)
Genitourinary system cancer	105 (3.07%)	87 (3.18%)	18 (2.62%)
Respiratory system cancer	114 (3.33%)	103 (3.77%)	11 (1.6%)
Other cancer^1^	121 (3.53%)	98 (3.58%)	23 (3.34%)
Cause of non‐cancer disease	834 (24.36%)	724 (26.47%)	110 (15.99%)
Nervous system disease	113 (3.3%)	101 (3.69%)	12 (1.74%)
Circulatory system disease	413 (12.07%)	369 (13.49%)	44 (6.4%)
Digestive system disease	17 (0.05%)	12 (0.044%)	5 (0.073%)
Respiratory system disease	115 (3.36%)	106 (3.88%)	9 (1.31%)
Diabetes mellitus	44 (1.29%)	33 (1.2%)	11 (1.6%)
Genitourinary system	33 (0.096%)	29 (1.06%)	4 (0.058%)
Other cause^2^	99 (2.89%)	74 (2.71%)	25 (3.63%)
Other causes of death^3^	172 (5.02%)	154 (5.63%)	18 (2.62%)

Other cancer^1^ include brain and other nervous system, gum and other mouth, in situ, benign or unknown behavior neoplasm, mesothelioma, miscellaneous malignant cancer, non‐melanoma skin.

Other cause^2^ include other infectious and parasitic diseases including HIV, septicemia, soft tissue including heart, state DC not available or state DC available but no COD, symptoms, signs and ill‐defined conditions.

Other causes of death^3^ include accidents and adverse effects, congenital anomalies, homicide and legal intervention, other cause of death.

**TABLE 2:** Population characteristics of persons diagnosed with penile squamous cell carcinoma (exclude other cancers) in the Surveillance, Epidemiology, and End Results Program (SEER) (1992–2018)

Characteristics	Overall US (*n* = 2201)	Non‐hispanics US (*n* = 1654)	Hispanics US (*n* = 547)
Age (year)			
Mean	64.92	67.29	57.73
Median (25th–75th percentile)	67.5 (57.5–77.5)	67.5 (57.5–77.5)	57.5 (47.5–72)
<40	115 (5.2%)	35 (2.1%)	80 (14.6%)
40–50	248 (11.3%)	137 (8.3%)	111 (20.3%)
50–60	403 (18.3%)	298 (18.0%)	105 (19.2%)
60–70	522 (23.7%)	419 (25.3%)	103 (18.8%)
70–80	499 (22.7%)	413 (25.0%)	86 (15.7%)
>80	414 (18.8%)	352 (21.3%)	62 (11.3%)
Year of diagnosis			
1992–1996	337	274	63
1997–2001	337	264	73
2002–2006	379	283	96
2007–2011	483	358	125
2012–2017	665	475	190
Median survival time (month) (25th–75th percentile)	67.98 (13–100.5)	68.32 (14–103)	66.84 (12–93%)
Mean survival time (month)	66.47	67.82	62.36
Median age at death (year)	72	72	67.5
3‐year mortality rate	711 (32.3%)	551 (33.31%)	160 (29.25%)
5‐year mortality rate	865 (39.3%)	679 (41.05%)	186 (34%)
Died from all causes	1205 (54.75%)	969 (58.59%)	236 (43.14%)
Died from Penile cancer	449 (20.4%)	336 (20.31%)	113 (20.66%)
Died from other disease	645 (29.3%)	536 (32.41%)	109 (19.93%)
Cause of other cancer	101 (4.59%)	78 (4.72%)	23 (4.2%)
Leukemia	3 (0.014%)	3 (0.018%)	0 (0%)
Digestive System cancer	4 (0.018%)	3 (0.018%)	1 (0.018%)
Genitourinary system cancer	14 (0.063%)	9 (0.054%)	5 (0.091%)
Respiratory system cancer	18 (0.082%)	17 (1.03%)	1 (0.018%)
Other cancer^1^	62 (2.8%)	46 (2.78%)	16 (2.9%)
Cause of Non‐cancer disease	544 (24.72%)	458 (27.69%)	86 (15.72%)
Nervous system disease	80 (3.63%)	69 (4.17%)	11 (2.01%)
Circulatory system disease	272 (12.36%)	234 (14.15%)	38 (6.95%)
Digestive system disease	9 (0.041%)	7 (0.042%)	2 (0.037%)
Respiratory system disease	71 (3.23%)	65 (3.93%)	6 (1.1%)
Diabetes mellitus	29 (1.32%)	20 (1.21%)	9 (1.65%)
Genitourinary system	23 (1.04%)	21 (1.27%)	2 (0.037%)
Other cause^2^	60 (2.73%)	42 (2.54%)	18 (3.29%)
Other causes of death^3^	111 (5.04%)	97 (5.86%)	14 (2.56%)

Other cancer^1^ include brain and other nervous system, gum and other mouth, in situ, benign or unknown behavior neoplasm, mesothelioma, miscellaneous malignant cancer, non‐melanoma skin.

Other cause^2^ include other infectious and parasitic diseases including HIV, septicemia, soft tissue including heart, state dc not available or state dc available but no cod, symptoms, signs and ill‐defined conditions.

Other causes of death^3^ include accidents and adverse effects, congenital anomalies, homicide and legal intervention, other cause of death.

### 2.3 Statistical analysis

We analyze penile squamous cell carcinoma incidence rates and mortality distribution of penile cancer patients by the following race and ethnicity cohorts: White men, Black men, Asian /Pacific Islander (API) men, American Indian/Alaska Native (AIAN) men, and Spanish‐Hispanic‐Latina men (called Hispanic for convenience).

We calculate age‐adjusted incidence rates (per million individuals) between 1992 and 2016 stratified by race. SEER*Stat software (version 8.3.9) is used to perform statistical calculations. The study period is averagely divided into five time periods because the annual incidence of penile squamous cell carcinoma is relatively low. Meanwhile, the age distribution of PSCC patients stratified by race is described, and the age has been divided into <40, 40–50, 40–60, 60–70, 70–80, and >80.

To remove the effect of other cancers (except PSCC) on the death of patients with PSCC, we analyze the distribution and changing trends of causes of death stratified by race in patients with primary penile cancer (excluding other cancers) and penile cancer (not excluding other cancers). The cause of death of patients with penile cancer is analyzed, and the percentage of patients with different causes of death in total patients is also presented. Three‐ and 5‐year mortality rates for different age groups stratified by race (non‐Hispanics and Hispanics) are calculated. The Kaplan–Meier method with the log‐rank test is performed to evaluate overall survival and cancer‐specific survival between races (White, Black, Hispanic, and other race).

Two‐sample *t‐tests* and Pearson's chi‐square tests are performed for continuous variables and categorical variables, respectively. The *p* < 0.050 is recognized as significant. The SPSS 22.0 (IBM Corp) and R version 3.6.3 are utilized for all statistical analyses.

## 3 RESULTS

### 3.1 Incidence of penile cancer and age distribution

**FIGURE 1:**
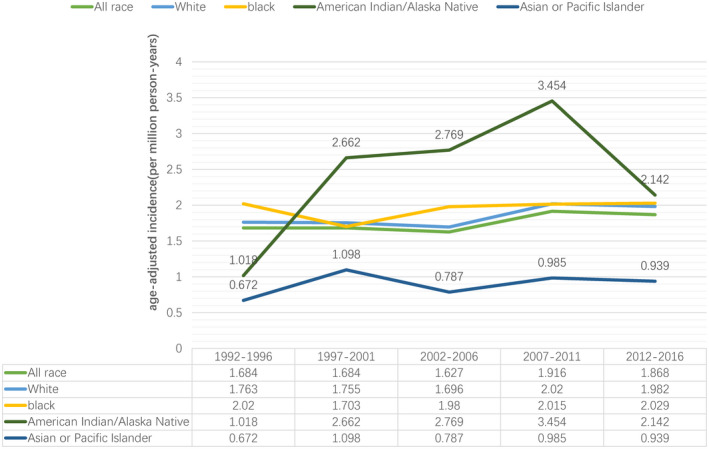
The age‐adjusted incidence of penile squamous cell carcinoma is illustrated according to the era of diagnosis stratified by race 338 × 190 mm (600 × 600 DPI)

Our analysis of the SEER 13 Registries for 1992–2018 includes 3423 male patients with penile cancer, while there are 2201 patients only with penile cancer (exclude other cancers). The average annual incidence of penile cancer for 1992–2016 is 0.351, 0.369, 0.398, 0.482, and 0.179 for all races, White, Black, AIAN, and API, respectively. AIAN has the highest average annual incidence, while the API has the lowest. Figure 1 illustrates the age‐adjusted incidence rates of penile cancer of different races by the era of diagnosis. The penile cancer incidence of White has a slight rise (Annual percent change [APC] = 0.647, 95% confidence interval [CI] = 0.036–1.263; *p* = 0.039), while regular changes of incidence fail to be observed in other races. A relatively lower incidence of API is shown at each period.

**FIGURE 2:**
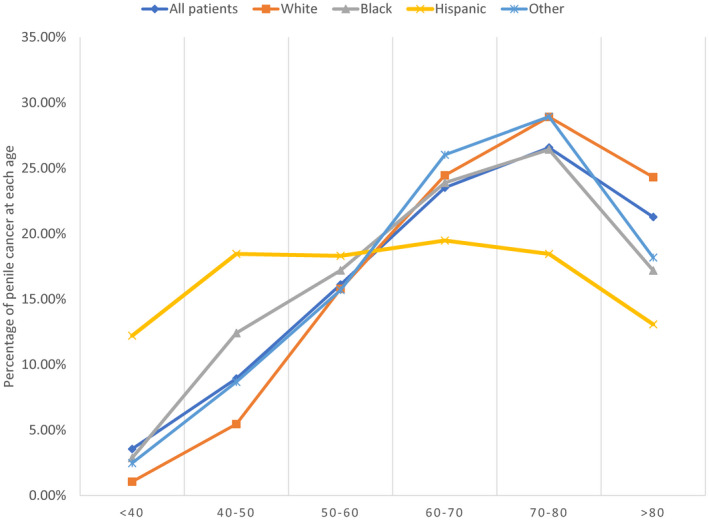
The incidence of penile squamous cell carcinoma is illustrated according to age decade at diagnosis stratified by race

**FIGURE 3:**
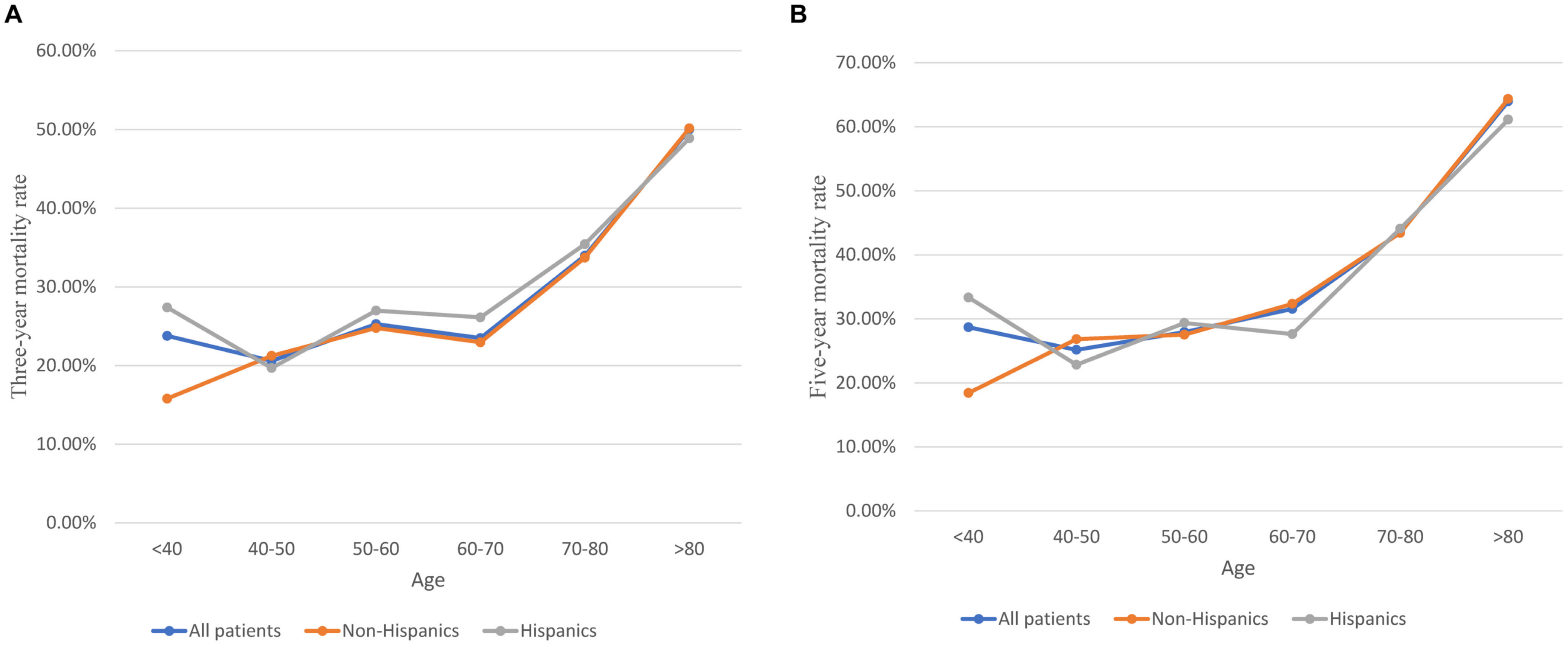
The 3‐ and 5‐year mortality rate are illustrated according to age decade at diagnosis stratified by race; (A): 3‐year mortality rate, (B) 5‐year mortality rate

Figure 2 demonstrates the age distribution of penile cancer of different races using a percentage of the total. The number of patients except for Hispanics with penile cancer have gradually increased under 80 years of age. Patients of all races except Hispanics aged 70–80 accounted for the highest percentage. (26.58% for all races, 28.91% for White, 26.43% for Black, 28.93% for other races). Hispanic fails to obtain a similar trend of age distribution, whereas a relatively higher percentage of young penile cancer patients (age <40 and age 40–50) are observed compared to other races. For various age groups, Figure 3 reveals a 3‐ and 5‐year mortality rate of all patients, non‐Hispanics and Hispanics. The 3‐ and 5‐year mortality rates have a significant upward trend among patients over 70 years old. Compared to the age group of 60–70 years, The extension of increase reaches 44.57% and 37.94% for 3‐ and 5‐year mortality rate, respectively, in the age group of 70–80; while the value is 113% and 103% in the age group of >80. For overall cases, patients in the age group of >80 are obtained the highest mortality rate both for 3‐year (50%) and 5‐year (63.93%). Similar results are observed in the race of Non‐Hispanics and Hispanics.

### 3.2 Distribution and changing trends and of the cause of death

Among all deaths occurring in the study period, only 28% of patients die from penile cancer. (Figure 5A) Even after excluding patients with other cancers, the ratio only increases to 37%. (Figure 5B) The most common cause of death in penile cancer patients is the non‐cancer disease (41%, Figure 5A), of which circulatory system disease is the most significant proportion (49.52% of non‐cancer disease). The ratio of death of non‐cancer disease patients have increased slightly when regarding patients with only PSCC (45% of all patients; Figure 5B). For all penile cancer patients, we do not obtain statistical differences on patients who died from penile cancer between non‐Hispanics and Hispanics (15.69% vs.19.33% of all patients before excluding other cancers; 20.31% vs. 20.66% of all patients after excluding other cancers; all *p* > 0.05). However, a more significant proportion of deaths from non‐cancer disease occurs in non‐Hispanics compared to Hispanics. (26.47% vs.15.99% of all patients before excluding other cancers; 27.69% versus 15.72% of all patients after excluding other cancers; *p* < 0.001). Therefore, the death cause of penile cancer is more relatively common in Hispanics. (42% of death Hispanics before excluding other cancers; 48% of death Hispanics after excluding other cancers; Figure 5E,F).

The proportion of patients who died of PSCC significantly increased from 21.17% to 41.3% for calendar 1992–2001 and 2012–2017, respectively, in PSCC patients (*p* < 0.001). (Table 3) We also obtain a similar result when we exclude patients without previous malignant tumors. (Table 3) In contrast, a prominent decrease in the ratio of patients who died of non‐cancer disease ranges from 46.92% to 30.75% for calendar 1992–2001 and 2012–2017, respectively, in PSCC patients. Of patients without previous malignant tumors, this change ranges from 53.33% to 30.73% (*p* < 0.001). (Table 3).

**TABLE 3:** Distributed of cause of death of penile squamous cell carcinoma for calendar 1992–2001, 2002–2011, and 2012–2017

Cause of death		Year of diagnosis		*p*‐value
	1992–2001	2002–2011	2012–2017	
PSCC (*N*, %)	893 (100%)	801 (100%)	322 (100%)	<0.001
Died from PSCC	189 (21.17%)	240 (30.03%)	133 (41.3%)	
Died from other cancer	199 (22.28%)	186 (23.2%)	63 (19.57%)	
Died from non‐cancer disease	419 (46.92%)	316 (39.4%)	99 (30.75%)	
Other cause^1^	86 (9.63%)	59 (7.37%)	27 (8.38%)	
PSCC (excluding other cancer) (*N*, %)	540 (100%)	460 (100%)	205 (100%)	<0.001
Died from PSCC	150 (27.78%)	192 (41.74%)	107 (52.2%)	
Died from other cancer	46 (8.5%)	37 (8.04%)	18 (8.78%)	
Died from non‐cancer disease	288 (53.33%)	193 (41.96%)	63 (30.73%)	
Other cause^1^	56 (10.37%)	38 (8.26%)	17 (8.29%)	

*Note*. Other cause: patients died of non‐disease factors.

Abbreviation: PSCC, penile squamous cell carcinoma.

### 3.3 Survival outcome and mortality rate of PSCC patients by race

**FIGURE 4:**
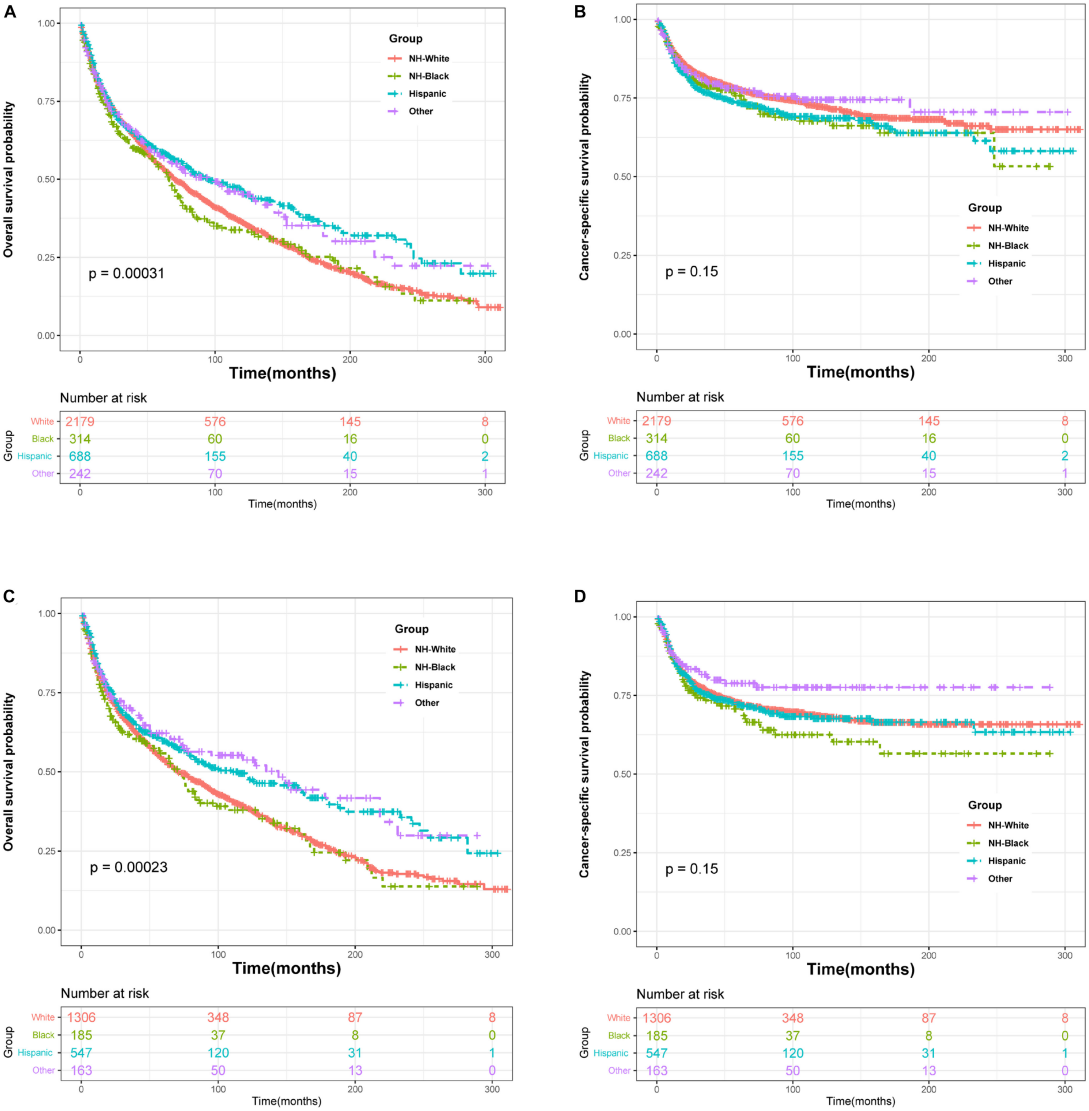
Overall survival and cancer‐specific survival for patients who were diagnosed with penile squamous cell carcinoma stratified by race: (A, B) for patients with penile squamous cell carcinoma; (C, D) for patients with only penile squamous cell carcinoma (exclude other cancer) 450 × 450 mm

The median and mean age at diagnosis of the non‐Hispanics are older than Hispanics. (72 vs. 62 for median age; 68.83 vs. 59.58 for mean age; all *p* < 0.001) (Table 1) Similar results are observed when just reserving patients with penile cancer (Table 2). Meanwhile, the median age at death of non‐Hispanics is also higher than those of Hispanics (72 vs. 57.5). The 3‐ and 5‐year mortality rates of penile cancer patients are 31.87% and 40.26%, respectively. When only considering penile cancer patients, the values are 32.3% and 39.3%. No significant changes are observed when excluding patients with other cancers. Non‐Hispanics patients show a higher mortality rate for 3‐ and 5‐year compared to Hispanics (32.36% vs. 29.94% for 3‐year; 41.55% vs 35.17% for 5‐year; *p* < 0.001). (Table 1) In addition, the survival curves confirm this conclusion. Figure 3 reveals overall survival and cancer‐specific survival between races in patients with penile cancer and penile cancer without other cancers. Hispanics are displayed better overall survival compared with non‐Hispanic (NH) White and non‐Hispanic (NH) Black patients. (All *p* < 0.001) (Figure 4 A.C.). However, statistical differences in CSS by race are failed to observe (All *p* > 0.05) (Figure 4B,D).

**FIGURE 5:**
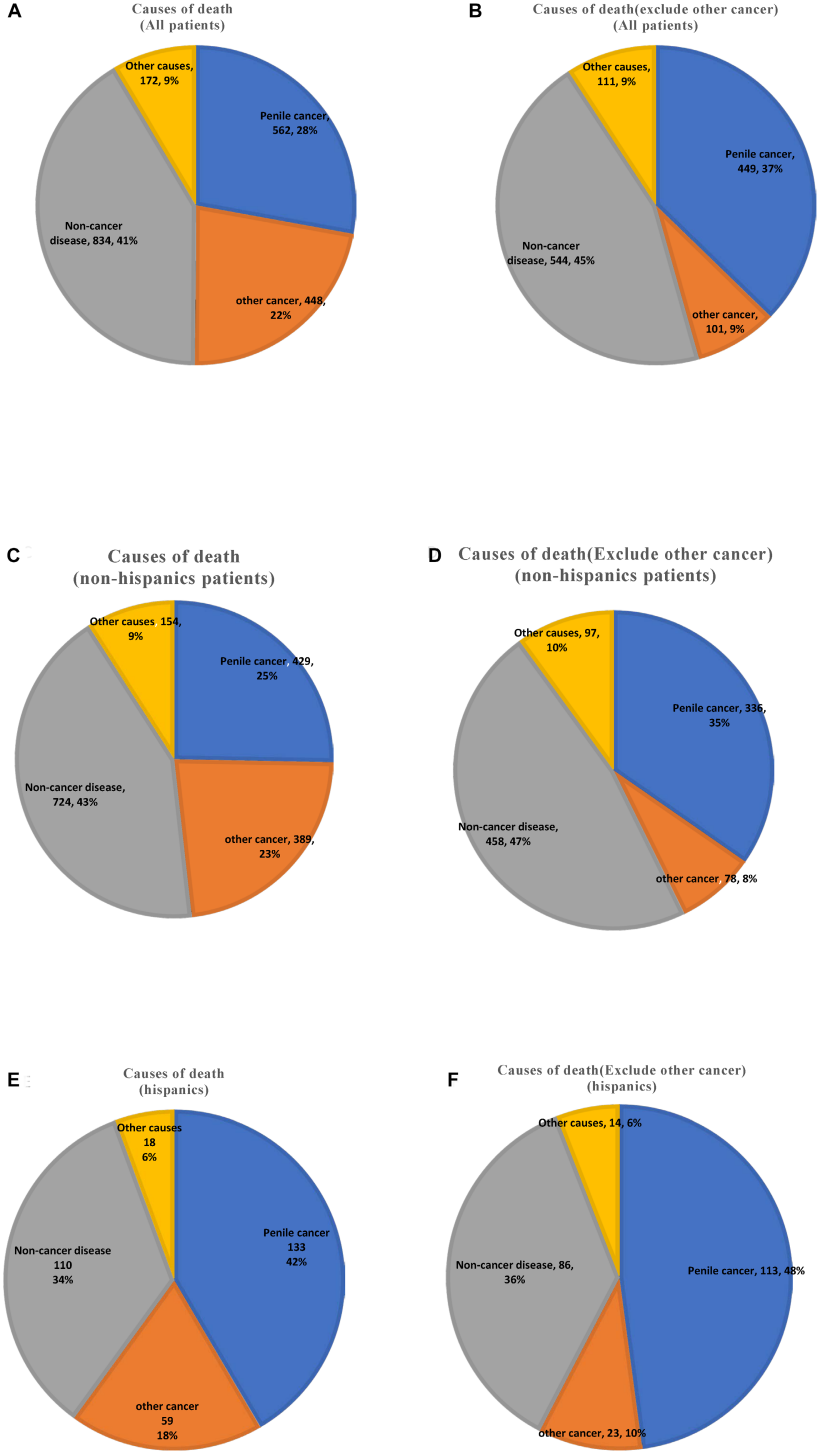
Distribution of causes of death stratified by race: (A, B) for all patients; (C, D) for non‐Hispanics patients; (E, F) for Hispanics patients; (A, C, E) for patients with penile squamous cell carcinoma; (B, D, F) for patients with only penile squamous cell carcinoma (exclude other cancer)

## 4 DISCUSSION

Several studies focusing on the incidence of PSCC from different countries have been performed. An interesting phenomenon is that the incidence rate in developed countries is much lower than that in developing countries.^3^, ^4^ However, the incidence rate still varies intensely, even concentrating on the population from the same country. A study based on the SEER database, whose data is from 39 population‐based cancer registries covering approximately 83% of the U.S. population, showed an annual average, the age‐adjusted incidence rate of 0.81 per 100,000 men.^5^ However, the incidence in our study with 13 registries is just only 0.351. Simultaneously, the differences in PSCC incidence rates between races have always been an interesting and a worth exploring issue. For example, the average age‐adjusted annual incidence of penile SCC of Hispanics is 72% higher than non‐Hispanics in the population of U.S. from 1998 to 2003.^5^ Similarly, our study investigates the incidence rate within 25 years in the U.S. and found that American Indian/Alaska Native men have the highest average age‐adjusted annual incidence of penile SCC, followed by Black, White, and Asian /Pacific Islander. What is noteworthy is that some different results about the incidence by race are proposed, and it might be due to the effect of some other factors like regions and living habits.^7^, ^18^ However, worldwide geographic and ethnic variation in penile SCC incidence is evident and convincing, and the cause of this result still needs more prospective studies to provide persuasive shreds of evidence. Interestingly, the trend of incidence by era has remained relatively stable in the period of 1992–2016, but a slightly increasing trend is observed in the white men.

Previous literature demonstrated that the most common age of presentation in penile SCC patients is between 50 and 70 years.^3^, ^17^ Nevertheless, patients aged 70–80 years predominate absolute and proportional incidence in this study. This phenomenon might be driven by improved medical treatment and the trend of the aging population. The increase in natural life expectancy made older patients the mainstay. Meanwhile, before the age of 80, the incidence rate of all patients except Hispanics is showing a gradual upward trend. We usually consider penile SCC a disease associated with age; namely, old patients might be more likely to increase the risk of penile SCC. However, the incidence trend related to age is not prominent in Hispanics. It might be the cause of other more effective interference factors such as human papilloma virus (HPV) infection and neonatal circumcision.^3^, ^5^, ^9^

We also investigate the distribution of 3‐ and 5‐year mortality rates of penile SCC patients in different age groups. If we treat the age group of 60–70 as a control, we could observe a significantly increasing both 3‐ and 5‐year mortality rate in all races. The percentage increase is 44.57% and 37.94% for 3‐ and 5‐year mortality rates, respectively, in the age group of 70–80, while the value is 113% and 103% in the age group of >80. Usually, in the population without penile SCC, a relatively higher mortality rate is more likely to be observed in older people, especially those with advanced age in comparison to those youthful.^19^, ^20^ Therefore, we cannot affirmatively assert that the mortality rate that increased with age is due to penile SCC. However, significantly increased mortality should not be ignored, and more meticulous care measures or appropriate treatment are a noteworthy issue for those with age over 70.

When regarding the distribution of cause of death in penile SCC patients, we interestingly find the most common cause of death is the non‐cancer disease (41% of all dead patients), but not penile SCC (28% of all dead patients). Similar results are still observed after excluding patients with other cancers. Furthermore, for non‐cancer disease, circulatory system disease is the most prominent part (49.52% of non‐cancer disease before excluding other cancers; 50% of non‐cancer disease after excluding other cancers). The circulatory system disease in this study included five categories: aortic aneurysm and dissection, atherosclerosis, diseases of heart, hypertension without heart disease, and other diseases of arteries, arterioles, capillaries. With the development of medicine, the treatment for some diseases with high mortality, including cancer, has been developing and improving.^21^ Chronic diseases such as hypertension, diabetes, and heart disease have been gradually the leading risk factor for global mortality.^22^, ^23^ Considering the natural increase in human lifespan, patients are generally diagnosed with penile SCC at older ages that we have discussed before in the previous content. Therefore, it seemed more reasonable that penile SCC patients are likely to be accompanied by other diseases, and these diseases played an increasingly important role in mortality.

Interestingly, we found that the proportion of patients who died of PSCC has increased significantly in recent years, and the proportion has exceeded that of the non‐cancer disease for the calendar of 2012–2017 (Table 3). Several previous studies pointed out that the tumor stage of PSCC was one of the most crucial prognostic factors.^11^, ^15^, ^24^, ^25^, ^26^ For instance, patients with a metastasizing malignant tumor were accompanying distinctly shorter survival time and higher recurrence rate.^25^, ^26^ In this study, the percentage of PSCC patients classified with localized stage were shown a decline ranging from 65.08% to 52.76% for the calendar of 1992–2001 and 2012–2017, respectively. Simultaneously, a relatively significant increase was observed in PSCC patients with advanced stages, including regional (31.23%–39.67%) and distant (3.7%–7.57%). This increasing trend in the advanced stage of PSCC patients might account for the analogous increasing trend in the proportion of patients who died of PSCC by era.

Several previous studies suggested the existence of different survival outcomes between various races. For instance, A study by *Sharma* et al. showed a poorer overall survival in Black penile SCC when compared with white patients.^18^ Analogously, of patients with PSCC in the United States, African‐American men were observed a worse survival outcome than that of Caucasian American men.^1^ While in this study, we perform survival curves and find that Hispanics have better overall survival than NH‐White and NH‐Black men (*p* < 0.001). However, when regarding PSCC‐specific survival as an endpoint, statistical differences in survival outcomes between races are failed to obtain. (*p* = 0.15) It is worth noting that we found no statistical differences in the distribution of cause of death from penile cancer between Non‐Hispanics and Hispanics. (Tables 1 and 2, *p* > 0.05) In addition, we have confirmed non‐cancer disease contributed the most of cause of death in penile SCC patients, and significant differences in the distribution of cause of non‐cancer disease are observed between Hispanics and Non‐Hispanic men (Tables 1 and 2; *p* < 0.05). Therefore, the discrepancies in overall survival between races might be the effect of non‐cancer disease instead of penile SCC.

Another interesting finding in the study is that we did not gain statistical differences in 3‐ and 5‐year mortality rates when we compared two types of the population––one with penile SCC and one with penile SCC excluding other cancer (*p* > 0.05). There is still no definite conclusion about the impact of past cancer history on the survival of existing cancers.^27^, ^28^, ^29^ A study focusing on 20 types of primary solid tumors from the SEER database suggested that patients with a prior cancer history showed similar overall survival to those without prior cancer.^28^ Further studies to confirm these results are still needed.

Our study is a population‐based analysis based on the SEER database, which is of extremely high quality in terms of accuracy and completeness. However, some limitations are still noteworthy in this study. First, variables like HPV status, circumcision status, and smoking behavior that might be of interest are not available in the SEER database. Secondly, considering the rarity of the disease, we have to classify some races into one category when we investigated the difference between races. For example, we have defined AIAN and API as other races when survival analysis is performed, and we just analyzed two general types of races, including Non‐Hispanics and Hispanics, when describing the distribution of cause of death. Furthermore, after excluding other cancers, there is still a small part of patients dying from other cancers. The primary reason for this result might be the cancer diagnosed after penile SCC would not be recorded in the SEER database, and some cancers are difficult to detect until the death appears.

## 5 CONCLUSION

The current study, using population‐level data from the SEER database, gets insight into penile squamous cell carcinoma from the trend of incidence, age distribution, distribution and changing trend of the cause of death, and survival outcome. Racial disparities are also explored. These results will be important for patient counseling and promotion for changing practice patterns. Notably, non‐cancer disease, especially circulatory system disease, is the more common cause of death than penile squamous cell carcinoma. However, the proportion of patients who died of penile squamous cell carcinoma has a relatively significant increase in recent years. The increasing trend in advanced stage of PSCC patients might account for this change.

## CONFLICT OF INTEREST

There were no conflicts of interest in this study.

## Author contribution

Xiangpeng and Luyao Chen contributed equally to this work and should be considered co‐first authors. All authors contributed to this article with: (1) Data analysis and collation; (2) drafting the article or revising it; (3) agreement to be accountable for all aspects of the work.

## Ethical approval statement

The data from SEER is publicly available and de‐identified. Consent is not requested.

## Data Availability

Data sharing is not applicable to this article as no new data were created or analyzed in this study.
